# Supplementary material for: A Risk Stratification System in Myeloma Patients with Autologous Stem Cell Transplantation
Source: Cancers (Basel). 2024 Mar 11;16(6):1116. doi: 10.3390/cancers16061116 (PMC10969019; doi:10.3390/cancers16061116)
Supplement: Supplementary file 1 [file cancers-16-01116-s001.zip › cancers-2882981-supplementary.pdf]

ATM4S: A Staging System for Assessing the Prognostic Benefits of Autologous Stem Cell Transplantation in Patients with Multiple Myeloma

Supplement

Catalog

Supplemental Methods 2
- Gene expression profiling and translocation prediction: 2
- Triple color fluorescence in situ hybridization: 3
- Data collection 3
- Data correction: 3
- Primary endpoints and secondary endpoints: 4
- Statistical analysis 4
  - Missing data: 4
  - Continuous variable and categorical variable transformation 4
  - Training set and validation set 4
  - Survival analysis 5
  - Variable selection for stage system 5
  - Stage system construction 5
  - Proportional hazards assessment 5
  - Assessment of model generalization performance 6
  - Comparison with R2-ISS system 6
  - Validation in patients diagnosed in three time periods 6
  - ATM4S combined with FISH and GEP scores 6

Supplemental Tables 6
- Supplemental Table S1. Data missingness statistics 6
- Supplemental Table S2. Baseline information of original data and data after imputation 7
- Supplemental Table S3. Criteria for discretizing continuous variables into categorical variables 9

Supplemental Table S4. Univariate Cox in training set 10
Supplemental Table S5. Multivariate Cox in training set 13
Supplemental Table S6. C-indexes of cox models as variables added in. 15
Supplemental Table S7. Clinical trials from the 5259 cohort. 15
Supplemental Table S8. Cox models' c-index of variables in ATM4S combine with GEP scores and chromosome translocation predicted by GEP in 2507 ASCT MM subset. 16
Supplemental Table S9. Cox models' c-index of variables in ATM4S combine with 17p del in 1019 ASCT MM subgroup 17
Supplemental Table S10. Cox models' c-index of variables in ATM4S combine with 13q del in 459 ASCT MM subgroup 18
Supplemental Table S11. Cox models' c-index of variables in ATM4S combine with 1p del and 1q gain in 1154 ASCT MM subgroup 18
Supplemental figures 20
Supplement Figure S1. Survival curves of variables present in < 20% high risk myeloma. 20
Supplement Figure S2. 4-stage system by k-adaptive partitioning and adjustment. 21
Supplement Figure S3. Assessment of model generalization (Overall survival). 22
Supplement Figure S4. ATM4S in different clinical trial subsets. 23
Reference 24

Supplemental Methods

Gene expression profiling and translocation prediction:

Plasma cell purifications and gene expression profiling (GEP) were carried out utilizing the Affymetrix U133Plus2.0 microarray platform (Santa Clara, CA) in accordance with well-established protocols (1, 2). The identification of t (4;14) and t(14;16) chromosome translocations was determined based on the presence of GEP expression "Spikes" for *FGFR3* and/or *NSD2*, and *MAF*, respectively. Additionally, classification into the MS or MF molecular subgroups, as reported by Zhan et al., 2006, was considered.

Triple color fluorescence in situ hybridization:

To detect deletion of 17p, we utilized the interphase-FISH procedure as previously described(3-5). The upper limit of normal plus three standard deviations (SD) for TP53 deletions, based on FISH studies conducted on normal bone marrow mononuclear cells, was found to be below 10%(6). Hence, we established the probe sets' background cutoff level at 10%.

The interphase-FISH procedure employed in this study for 1q gain has been previously documented(3). Mononuclear cells obtained from bone marrow aspirates were prepared as cytospin preparations and subjected to Ficoll separation. The cells were fixed with ethanol, followed by hybridization of probes. AMCA-labeled antibodies targeting κ or λ immunoglobulin light chains (Vector Laboratories, Burlingame, CA) were used during the hybridization process. The resulting slides were stored at -20°C until FISH analyses were conducted. In each patient, a minimum of 100 clonal plasma cells (predominantly 100 cells) were assessed for interphase FISH signals. Gain/amplification was considered present when at least 3 copies were detected in at least 20% of clonal plasma cells. To investigate the impact of Amp1q21 on clinical outcomes and differentiate between categories, Amp1q21 was classified into two groups: (1) 3 copies of 1q21 (with less than 20% of clonal plasma cells showing at least 4 copies) and (2) at least 4 copies of 1q21 (with at least 20% of clonal plasma cells showing at least 4 copies).

Data collection

From 1989 to 2022 5749 MM patients received ASCT at UAMS. After filtering overall survival, progression free survival and time from transplant to diagnosis, we obtained a cohort of 5259 for the present studies (Figure 1). Thirty demographic and clinical variables were collected and included in this analysis. They included, age at transplant date, gender, race, isotype, serum light chain type, urine light chain type, iron, transferrin, ferritin, plasma cell percentage, albumin, B2M, LDH, creatinine, CRP, hemoglobin, platelets, monocytes, lymphocytes, serum M protein, urine M protein, Calcium, BMI, serum glucose, cholesterol, triglycerides, HDL and LDL. Most of the clinical data were collected at the myeloma diagnosis date at UAMS. In addition, we also collected genetic data including gene expression profiling scores (GEP70, GEP80, SKY92, and proliferation index), high-risk chromosomal translocations (t(4;14), t(14;16), t(14;20) predicted by GEP, and FISH data (del 1p, gain 1q, amp1q21, del 17p del, del 13q). Subset information of the 5259 is shown in Figure 1.

Data correction:

ISS stages were re-calculated using ALB and B2M levels(7). R2-ISS(8) stages were calculated based on the clinical and chromosome abnormality data.

Primary endpoints and secondary endpoints:

Start time point was set as the transplant date. Considering the medium follow-up time is 57 months (Q1: 23.4 months, Q3: 112.5 months), progression free survival was set as primary event endpoints. The progression events after 2006 were made according to IMWG standard(9) (https://www.myeloma.org/resource-library/international-myeloma-working-group-imwg-uniform-response-criteria-multiple). Those progression events before 2006 were defined by myeloma physicians according to clinical observation and disease status.

Statistical analysis

Missing data:

For clinical data, we computed the rates of missing values for each variable (Supplemental Table S1). Urine light chain types and LDL were absent in over 50% of the cases and therefore excluded from the model construction. The “mice” package (version 3.15.0) was used for missing completely at random test and the assumption of Missing Completely at Random (MCAR) holds. Multiple imputation was performed using the Random Forest method. Baseline information of original data and data after imputation are listed in Supplemental Table S2. For cytogenetic data, we only analyzed complete data.

Continuous variable and categorical variable transformation

All continuous clinical variables were transformed into categorical variable according to clinical standard. For GEP scores (GEP70(10), GEP80(11), PI(2) and SKY92(12)), they were transformed into a binary as originally described (Supplemental Table S3).

Training set and validation set

70% of the samples were randomly allocated into a training cohort and the remaining 30% used as a validation cohort (“caret” package, version 6.0-88 in R4.0.5) arranged by patient identification. Baseline information of training and validation cohorts are listed in Table 1.

Survival analysis

CBCgrps(13) (version 2.8.2) was used for baseline Table . We used R4.0.5 software for statistical analysis. “survival” (version 3.2-10) and “survminer” (version 0.4.9.999) were used for survival analysis and visualization. Univariate Cox was used to evaluate the prognostic role of each variable (Supplemental Table S4). Those variables with $p<0.05$ were put into multivariate Cox (Supplemental Table S5).

Variable selection for stage system

We selected those variables with $p<0.05$ in univariate Cox. To generate an evenly distributed system, variables present in less than 20% of poor prognosis cases were excluded. Those excluded variables included platelets, calcium and isotype (Supplemental Figure S1). We started from the ISS stage system and added variables in a stepwise manner. Harrel’s c-index was used to evaluate the effect of adding additional variables to ISS. As more variables were added into the system the curves of c-index plateaued (Figure 2A-C, supplementary Table S6), leading us to select the seven variables that had the greatest effect on the c-index for model construction.

Stage system construction

We then proceeded to assign weighted scores to each variable, subsequently enabling the computation of an absolute risk score for each patient. The risk scores were further partitioned into distinct stages based on the magnitude of the risk score. Multivariate Cox was performed using the seven variables. We calculated a risk score for each variable by normalizing “hazard ratio” (average hazard ratio of PFS and OS) of ISS stage III vs ISS stage I&II to 1 and selecting the value closest to an integer or an integer plus 0.5. Finally, the sum risk scores of each patient were calculated by addition. Then k-adaptive partitioning method was performed to determine score borders for each stage, making the difference in survival outcomes as large as possible between different stages. We start from the border of 4-stage system defined by k-adaptive partitioning and then adjust the border to make the distribution even (Supplemental Figure S2).

Proportional hazards assessment

We generated log-negative log plots using both OS and PFS based on the ATM4S risk groups in both the training set and validation set (Figure 2G, H; Figure 3C, D). This visual approach was employed to assess the proportional hazards assumption.

Assessment of model generalization performance

We also tested the OS and PFS calibration of ATM4S in the validation cohort. There is no significance difference in patients with the same stage of ATM4S in training and validation cohorts (Figure 3C-F, Supplementary Figure S3C-F).

Comparison with R2-ISS system

To make a comparison between ATM4S and R2-ISS, we derived a subset of 860 patients with both R2-ISS and ATM4S stage information. Harrel C-indexes were calculated for the two systems. Figure 4C shows the distribution of ATM4S stages and R2ISS stages. ATM4S had a higher c-index compared to R2-ISS (Figure 4D).

Validation in patients diagnosed in three time periods

As this study included patients receiving ASCT from 1989 to 2022, we separated the whole cohort into three chronological groups. Kaplan-Meier curve was used to evaluate ATM4S in three subsets (Figure 5).

ATM4S combined with FISH and GEP scores

We were interested to determine if the performance of the model could be improved with the addition of high-risk genetic variables. As shown in Figure 1, a subset of the 5259 have genetic information such as a subset with GEP, a subset with 1q and 1p del data, a subset with 13q del and a subset with 17p del subset. These subsets were further evaluated to see if ATM4S combine with cytogenetic information will have higher c-indexes (Supplemental Tables S8-S11).

Supplemental Tables

Supplemental Table S1. Data missingness statistics

Variables	Missing proportion
Sex	0
Race	0.018445
Isotype	0.01236
Light	0.014261
Transferrin	0.453889
Ferritin	0.289028

IRON	0.283514
Plasma cell percentage by aspiration	0.078532
Plasma cell percentage by biopsy	0.081955
Albumin	0.008176
B2M	0.029473
LDH	0.033847
Creatinine	0.015782
CRP	0.051911
Hb	0.01217
Platelets	0.01255
Monocytes	0.01217
Lymphocytes	0.008937
Serum M protein	0.036129
Urine M protein	0.046397
Ca	0.018254
BMI	0.456361
Serum glucose	0.017114
Cholesterol	0.191671
Triglycerides	0.183685
HDL	0.496863
LDL	0.509412
Time from diagnosis to ASCT	0
OS time	0
OS	0
PFS time	0
PFS	0
Age at ASCT	0

Abbreviation. B2M, Beta-2 Microglobulin; LDH, Lactate Dehydrogenase; CRP, C-Reactive Protein; Hb, Hemoglobin; BMI, Body Mass Index; HDL, High-Density Lipoprotein; OS, overall survival; PFS, progression free survival.

Supplemental Table S2. Baseline information of original data and data after imputation

	Original data	Imputated data
Variables	Total (n = 5259)	Total (n = 5259)
Sex, n (%)		
Female	2081 (40)	2081 (40)
Male	3178 (60)	3178 (60)

Age at ASCT, Median (Q1,Q3)	59.33 (51.66, 66.25)	59.33 (51.66, 66.25)
Isotype, n (%)		
Biclonal disease	11 (0)	11 (0)
Free light chain	927 (18)	936 (18)
Iga	1043 (20)	1051 (20)
Igd	65 (1)	65 (1)
Igg	2867 (55)	2905 (55)
igm	20 (0)	20 (0)
Nonsecretory	261 (5)	271 (5)
Light Chain types (serum), n (%)		
kappa	3152 (61)	3188 (61)
kappa + lambda	6 (0)	6 (0)
lambda	1800 (35)	1823 (35)
none	226 (4)	242 (5)
Iron, Median (Q1,Q3), µg/dL	72 (51.75, 97)	72 (52, 98)
Transferrin, Median (Q1,Q3), g/L	215 (179, 249)	214 (177, 248)
Ferritin, Median (Q1,Q3), µg/L	228.7 (96.85, 506.75)	238 (100.7, 538.1)
Plasma cell percentage by aspiration, Median (Q1,Q3), %	25 (7.5, 50)	25 (7.5, 50)
Plasma cell percentage by biopsy, Median (Q1,Q3), %	30 (7.5, 60)	30 (7.5, 60)
Albumin, Median (Q1,Q3), g/dL	3.9 (3.5, 4.3)	3.9 (3.5, 4.3)
B2M, Median (Q1,Q3), mg/L	3.1 (2.11, 5.3)	3.1 (2.13, 5.3)
LDH, Median (Q1,Q3), U/L	158 (128, 199)	158 (128, 199)
Creatinine, Median (Q1,Q3), mg/dL	1 (0.8, 1.3)	1 (0.8, 1.3)
CRP, Median (Q1,Q3), mg/dL	2.7 (0.45, 5.5)	2.5 (0.45, 5.5)
Hb, Median (Q1,Q3), g/dl	11.3 (9.8, 12.8)	11.3 (9.8, 12.8)
Platelets, Median (Q1,Q3), 10^3/µL	222 (170, 278)	221 (170, 278)
Monocytes, Median (Q1,Q3), %	8.9 (6.8, 11.7)	8.9 (6.85, 11.7)
Lymphocytes, Median (Q1,Q3), %	26.3 (18.48, 35.2)	26.3 (18.4, 35.2)
Serum M protein, Median (Q1,Q3), g/dL	1.5 (0.1, 3.6)	1.4 (0.1, 3.55)
Urine M protein, Median (Q1,Q3), g/L	0 (0, 508)	0 (0, 495)
Ca, Median (Q1,Q3), mg/dL	9.2 (8.8, 9.7)	9.2 (8.8, 9.7)
BMI, Median (Q1,Q3), kg/m2	27.99 (24.96, 31.85)	27.94 (24.81, 31.59)
Serum glucose, Median (Q1,Q3), mmol/L	100 (90, 119)	100 (90, 119)
Cholesterol, Median (Q1,Q3), mg/dL	173 (139, 207)	172 (138, 207)
Triglycerides, Median (Q1,Q3), mg/dL	141 (93, 211)	141 (93, 210)
HDL, Median (Q1,Q3), mg/dL	42 (33, 53)	42 (33, 53)
Time from MM diagnosis to ASCT, Median (Q1,Q3), mth	6.5 (4.17, 12.02)	6.5 (4.17, 12.02)
OS time, Median (Q1,Q3), mth	57 (23.4, 112.52)	57 (23.4, 112.52)
OS, n (%)		
0	1920 (37)	1920 (37)

1	3339 (63)	3339 (63)
PFS time, Median (Q1,Q3), mth	39.57 (15.17, 84.63)	39.57 (15.17, 84.63)
PFS, n (%)		
0	1630 (31)	1630 (31)
1	3629 (69)	3629 (69)

Abbreviation. Q1, first quantile; Q3, third quantile; B2M, Beta-2 Microglobulin; LDH, Lactate Dehydrogenase; CRP, C-Reactive Protein; Hb, Hemoglobin; BMI, Body Mass Index; HDL, High-Density Lipoprotein; OS, overall survival; PFS, progression free survival.

Supplemental Table S3. Criteria for discretizing continuous variables into categorical variables

Variables	Border
Time from MM diagnosis to ASCT, mth	long: >12
IRON, μg/dL	Male: high: >170; low: <60 Female: high: >150; low: <50
Transferrin, g/L	high: >360; low: <200
Ferritin, μg/L	Male: high: >336; low: <24 Female: high: >306; low: <11
Plasma cell percentage, %	high: >=60
Albumin, g/dL	low: <3.5
B2M, mg/L	high: >=5.5
LDH, U/L	high: >=190
Creatininine, mg/dL	high: >=2
CRP, mg/dL	high: >=8
Hb, g/dl	low: =<10
Platelets, 10^3/μL	low: <150
Monocytes, %	high: >8; low: <2
Lymphocytes, %	high: >40; low: <20
Serum M protein, g/dL	low: <3
Urine M protein, g/L	low: <0.3
Calcium, mg/dL	low: <8.5; high: >10.5
BMI, kg/m2	low: <18.5; high: >24.9
Serum Glucose, mmol/L	high: >=200
Cholesterol, mg/dL	low: <125; high: >200
Triglycerides, mg/dL	high: >=150
HDL, mg/dL	low: <40
Age, yr	young: <65

Abbreviation. B2M, Beta-2 Microglobulin; LDH, Lactate Dehydrogenase; Hb,

Hemoglobin; BMI, Body Mass Index; HDL, High-Density Lipoprotein; CRP, C-Reactive Protein.

Supplemental Table S4. Univariate Cox in training set

Variable	HR (PFS)	HR (PFS)	P val (PFS)	HR (OS)	HR (OS)	P val (OS)
Sex						
Female	Reference			Reference		
Male	1.156	[1.067, 1.252]	0.0003	1.158	[1.064, 1.259]	0.0006
Race						
White	Reference			Reference		
African	0.9022	[0.7946, 1.024]	0.112	0.9101	[0.7965, 1.04]	0.166
Others	0.6927	[0.4016, 1.195]	0.187	0.7172	[0.3965, 1.297]	0.272
Isotype						
Igg	Reference			Reference		
Free light chain	0.9831	[0.8858, 1.091]	0.7488	1.046	[0.9387, 1.167]	0.412
IgA	1.1923	[1.080, 1.317]	0.0005	1.253	[1.131, 1.389]	<0.0001
Non-secretory	0.9382	[0.7756, 1.135]	0.5114	1.055	[0.8667, 1.285]	0.592
Others	1.2925	[0.9871, 1.692]	0.0621	1.276	[0.9609, 1.695]	0.092
Light						
Kappa	Reference			Reference		
Lambda	1.1645	[1.063, 1.276]	0.0128	1.181	[1.085, 1.285]	0.000123
Others (Mainly none)	0.8477	[0.7687, 0.9347]	0.4567	1.047	[0.853, 1.286]	0.659416
U Light						
Kappa	Reference			Reference		
Lambda	1.1018	[1.005, 1.208]	0.0011	1.2292	[1.118, 1.351]	<0.0001
Others (Mainly none)	0.7914	[0.7171, 0.8734]	0.0009	0.8072	[0.728, 0.895]	<0.0001
Transferring						
Normal	Reference			Reference		
High	1.176	[0.7073, 1.956]	0.532	0.8173	[0.4707, 1.419]	0.717
Low	1.568	[1.449, 1.696]	<0.0001	1.6922	[1.5587,	<0.0001

					1.837]	
Ferritin						
Normal	Reference			Reference		
High	1.688	[1.560, 1.826]	<0.0001	1.751	[1.6134, 1.901]	<0.0001
Low	1.001	[0.771, 1.299]	0.996	1.011	[0.7708, 1.326]	0.936
Iron						
Normal	Reference			Reference		
High	1.539	[1.3044, 1.815]	<0.0001	1.538	[1.297, 1.825]	<0.0001
Low	1.076	[0.9865, 1.173]	0.0983	1.194	[1.091, 1.306]	0.0001
ALB						
Normal	Reference			Reference		
Low	1.238	[1.131, 1.356]	<0.0001	1.377	[1.254, 1.513]	<0.0001
B2M						
Normal	Reference			Reference		
High	1.506	[1.381, 1.643]	<0.0001	1.648	[1.506, 1.802]	<0.0001
ISS						
I	Reference			Reference		
II	1.073	[0.9782, 1.177]	0.135	1.132	[1.027, 1.248]	0.0124
III	1.549	[1.4093, 1.703]	<0.0001	1.73	[1.568, 1.909]	<0.0001
LDH						
Normal	Reference			Reference		
High	1.338	[1.231, 1.454]	<0.0001	1.49	[1.367, 1.624]	<0.0001
Creatinine						
Normal	Reference			Reference		
High	1.313	[1.180, 1.462]	<0.0001	1.541	[1.381, 1.719]	<0.0001
CRP						
Normal	Reference			Reference		
High	1.112	[0.9986, 1.239]	0.053	1.219	[1.089, 1.365]	0.0006
Hb						
Normal	Reference			Reference		
Low	1.372	[1.261, 1.493]	<0.0001	1.422	[1.303, 1.552]	<0.0001
Platelets						
Normal	Reference			Reference		
Low	1.82	[1.655, 2.001]	<0.0001	1.895	[1.718, 2.089]	<0.0001
Monocytes						

Normal	Reference			Reference		
High	1.188	[1.095, 1.288]	<0.0001	1.212	[1.113, 1.32]	<0.0001
Low	1.457	[1.093, 1.943]	0.0103	1.484	[1.103, 1.997]	0.0092
Lymphocytes						
Normal	Reference			Reference		
High	1.017	[0.9073, 1.140]	0.77	0.9657	[0.8559, 1.089]	0.570
Low	1.254	[1.150, 1.368]	<0.0001	1.3432	[1.2277, 1.47]	<0.0001
Serum M						
Normal	Reference			Reference		
High	1.034	[0.9519, 1.123]	0.429	0.9442	[0.8658, 1.03]	0.194
Urine M						
Normal	Reference			Reference		
High	1.136	[1.051, 1.228]	0.0013	1.212	[1.118, 1.315]	<0.0001
Ca						
Normal	Reference			Reference		
High	1.44	[1.2515, 1.656]	<0.0001	1.517	[1.314, 1.75]	<0.0001
Low	1.056	[0.9358, 1.192]	0.375	1.106	[0.975, 1.256]	0.375
BMI						
Normal	Reference			Reference		
High	0.9177	[0.8399, 1.003]	0.0571	0.8931	[0.8144, 0.9793]	0.0162
Low	0.7547	[0.4783, 1.191]	0.2264	0.7328	[0.4461, 1.2039]	0.2197
Serum glucose						
Normal	Reference			Reference		
High	1.249	[1.016, 1.535]	0.0346	1.378	[1.115, 1.704]	0.0030
Cholesterol						
Normal	Reference			Reference		
High	0.9347	[0.8544, 1.023]	0.1410	0.9141	[0.8321, 1.004]	0.0611
Low	1.2007	[1.0775, 1.338]	0.0009	1.245	[1.1125, 1.393]	0.0001
Triglycerides						
Normal	Reference			Reference		
High	1.134	[1.050, 1.226]	0.0014	1.122	[1.035, 1.216]	0.0054
HDL						

Normal	Reference			Reference		
High	1.201	[1.111, 1.298]	<0.0001	1.207	[1.113, 1.31]	<0.0001
Time from MM diagnosis to ASCT						
Short	Reference			Reference		
Long (>1 year)	1.857	[2.023, 1.959]	<0.0001	2.08	[1.904, 2.272]	<0.0001
Age at ASCT						
Young (<65)	Reference			Reference		
Old	1.215	[1.117, 1.322]	<0.0001	1.44	[1.319, 1.571]	<0.0001

Abbreviation. HR, Hazard Rratio; B2M, Beta-2 Microglobulin; LDH, Lactate Dehydrogenase; CRP, C-Reactive Protein; Hb, Hemoglobin; BMI, Body Mass Index; HDL, High-Density Lipoprotein;

Supplemental Table S5. Multivariate Cox in training set

Variables	HR (PFS)	95%CI (PFS)	P (PFS)	HR (OS)	95%CI (OS)	P (OS)
Age at ASCT	1.152	[1.058, 1.255]	0.0012	1.354	[1.238, 1.480]	<0.0001
Albumin low	1.068	[0.9675, 1.179]	0.1916	1.145	[1.033, 1.269]	0.0096
B2M high	1.165	[1.035, 1.312]	0.0114	1.152	[1.019, 1.303]	0.0242
Ca high	1.269	[1.097, 1.469]	0.0014	1.304	[1.122, 1.515]	0.0005
Ca low	0.903	[0.7953, 1.026]	0.1189	0.925	[0.8107, 1.056]	0.2513
Cholesterol high	0.948	[0.8647, 1.040]	0.2577	0.929	[0.8440, 1.023]	0.1342
Cholesterol low	1.019	[0.9070, 1.146]	0.7484	1.067	[0.9443, 1.205]	0.2987
Creatinine high	0.896	[0.7803, 1.028]	0.1177	1.032	[0.8952, 1.190]	0.6644
Ferritin high	1.336	[1.225, 1.456]	<0.0001	1.368	[1.251, 1.497]	<0.0001
Ferritin low	0.976	[0.7488, 1.271]	0.8547	0.952	[0.7221, 1.254]	0.7258
Serum glucose high	1.148	[0.9313, 1.415]	0.1959	1.225	[0.9879, 1.520]	0.0644

Hb low	1.048	[0.9502, 1.155]	0.3500	1.041	[0.9404, 1.151]	0.4415
HDL low	1.061	[0.9768, 1.152]	0.1602	1.070	[0.9818, 1.166]	0.1231
Iron high	1.184	[0.9985, 1.403]	0.0521	1.125	[0.9434, 1.341]	0.1900
Iron low	1.029	[0.9397, 1.127]	0.5361	1.113	[1.012, 1.223]	0.0274
Isotype free light chain	0.923	[0.8239, 1.034]	0.1651	0.960	[0.8533, 1.081]	0.5027
Isotype IgA	1.195	[1.079, 1.324]	0.0006	1.253	[1.127, 1.393]	<0.0001
Isotype non-secretory	0.933	[0.6503, 1.339]	0.7078	1.022	[0.7012, 1.489]	0.9110
Isotype Others	1.164	[0.8855, 1.531]	0.2761	1.155	[0.8662, 1.540]	0.3261
LDH high	1.270	[1.163, 1.387]	<0.0001	1.374	[1.255, 1.506]	<0.0001
Serum light lambda	1.081	[0.9944, 1.176]	0.0675	1.152	[1.056, 1.257]	0.0014
Serum light Others	1.081	[0.7412, 1.577]	0.6853	1.193	[0.8053, 1.767]	0.3788
Lymphocytes high	1.023	[0.9102, 1.150]	0.7014	1.027	[0.9075, 1.162]	0.6741
Lymphocytes low	1.108	[1.011, 1.214]	0.0288	1.137	[1.034, 1.250]	0.0079
Time from diagnosis to ASCT Long	1.869	[1.710, 2.044]	<0.0001	2.152	[1.963, 2.360]	<0.0001
Monocytes high	1.067	[0.9829, 1.159]	0.1210	1.075	[0.9862, 1.172]	0.1003
Monocytes low	1.165	[0.8700, 1.559]	0.3060	1.233	[0.9128, 1.665]	0.1722
Plasma cell percentage high	1.081	[0.9854, 1.187]	0.0990	1.024	[0.9287, 1.128]	0.6376
Platelets low	1.435	[1.295, 1.590]	<0.0001	1.411	[1.269, 1.569]	<0.0001
Sex male	1.141	[1.048, 1.242]	0.0024	1.118	[1.024, 1.222]	0.0133
Transferrin high	1.491	[0.8896, 2.498]	0.1295	1.169	[0.6669, 2.049]	0.5856
Transferrin low	1.309	[1.199, 1.428]	<0.0001	1.344	[1.226, 1.472]	<0.0001
Triglycerides high	1.067	[0.9827, 1.157]	0.1231	1.063	[0.9762, 1.158]	0.1596
Urine M protein high	0.990	[0.9065, 1.082]	0.8324	1.029	[0.9386, 1.128]	0.5448

Abbreviation. HR, Hazard Ratio, B2M, Beta-2 Microglobulin; LDH, Lactate

Dehydrogenase; CRP, C-Reactive Protein; Hb, Hemoglobin; BMI, Body Mass Index; HDL, High-Density Lipoprotein;

Supplemental Table S6. C-indexes of cox models as variables added in.

Variables used for cox model	C-index (PFS)	C-index (OS)
ISS	0.5535	0.5689
ISS+Diagnosis_to_ASCT	0.5999	0.6231
ISS+Diagnosis_to_ASCT+Ferritin	0.6219	0.6429
ISS+Diagnosis_to_ASCT+Ferritin+Transferrin	0.6287	0.6496
ISS+Diagnosis_to_ASCT+Ferritin+Transferrin+LDH	0.6334	0.6560
ISS+Diagnosis_to_ASCT+Ferritin+Transferrin+LDH+Age.at.ASCT	0.6351	0.6608
ISS+Diagnosis_to_ASCT+Ferritin+Transferrin+LDH+Age.at.ASCT+Sex	0.6357	0.6626
ISS+Diagnosis_to_ASCT+Ferritin+Transferrin+LDH+Age.at.ASCT+Sex+Lymphocytes	0.6366	0.6640

Abbreviation. ISS, the International Stage System; Diagnosis_to_ASCT, time from MM diagnosis to ASCT date; LDH, Lactate Dehydrogenase; Age.at.ASCT, age at ASCT date.

Supplemental Table S7. Clinical trials from the 5259 cohort.

Clinical Trial	Title	Number of participants
TT1	Phase II Study of Intensive Total Therapy For untreated or Minimally Treated patients With Multiple Myeloma	190
TT2	Total Therapy II - A Phase III Study for Newly Diagnosed Multiple Myeloma Evaluating Anti-Angiogenesis with Thalidomide and Post-Transplant Consolidation Chemotherapy	556
TT3a	A Phase 2 Study Incorporating Bone Marrow Microenvironment (ME) - Co-Targeting Bortezomib into Tandem Melphalan-Based Autotransplants with	292

	DT PACE for Induction/Consolidation and Thalidomide + Dexamethasone for Maintenance	
TT3b	Total Therapy 3B: An Extension of UARK 2003-33 Total Therapy 3: A Phase II Study Incorporating Bone Marrow Microenvironment (ME) – Co-Targeting Bortezomib into Tandem Melphalan-Based Autotransplants with DTPACE for Induction/Consolidation and Thalidomide + Dexamethasone for Maintenance	253
TT4	A Phase III Trial for Low-Risk Myeloma Ages 65 and Under: A Trial Enrolling Subjects to Standard Total Therapy 3 (S-TT3)	416
TT5	A PHASE II TRIAL FOR HIGH-RISK MYELOMA EVALUATING ACCELERATING AND SUSTAINING COMPLETE REMISSION (AS-CR) BY APPLYING NON-HOST-EXHAUSTING AND TIMELY DOSE-REDUCED MEL-80-VRD-PACE TANDEM TRANSPLANTS WITH INTERSPERSED MEL-20-VTD-PACE AND ALTERNATING VRD AND VMD MAINTENANCE	77
TT5b	A PHASE II TRIAL FOR HIGH-RISK MYELOMA EVALUATING ACCELERATING AND SUSTAINING COMPLETE REMISSION (AS-CR) BY APPLYING NON-HOST-EXHAUSTING AND TIMELY DOSE-REDUCED MEL-80-CFZ-TD-PACE TANDEM TRANSPLANTS WITH INTERSPERSED MEL-20-CFZ-TD-PACE WITH CFZ-RD AND CFZ-D MAINTENANCE	18
TT6	PHASE II TRIAL FOR PATIENTS NOT QUALIFYING FOR TT4 AND TT5 PROTOCOLS BECAUSE OF PRIOR THERAPY (NO PRIOR TRANSPLANT)	176

Abbreviation: TT, Total therapy.

Supplemental Table S8. Cox models' c-index of variables in ATM4S combine with GEP scores and chromosome translocation predicted by GEP in 2507 ASCT MM subset.

Variables in Cox model	c-index of OS	c-index of PFS
ISS+Diagnosis_to_ASCT+Ferritin+Transferrin+LDH+Age_at_ASCT+Sex	0.6692	0.6289
ISS+Diagnosis_to_ASCT+Ferritin+Transferrin+LDH+Age_at_ASCT+Sex+gep70	0.7069	0.6672
ISS+Diagnosis_to_ASCT+Ferritin+Transferrin+LDH+Age_at_ASCT+Sex+gep80	0.7032	0.6630
ISS+Diagnosis_to_ASCT+Ferritin+Transferrin+LDH+Age_at_ASCT+Sex+PI	0.7040	0.6635
ISS+Diagnosis_to_ASCT+Ferritin+Transferrin+LDH+Age_at_ASCT+Sex+Sky92	0.6992	0.6595
ISS+Diagnosis_to_ASCT+Ferritin+Transferrin+LDH+Age_at_ASCT+Sex+14;16	0.6701	0.6331
ISS+Diagnosis_to_ASCT+Ferritin+Transferrin+LDH+Age_at_ASCT+Sex+14;20	0.6707	0.6327
ISS+Diagnosis_to_ASCT+Ferritin+Transferrin+LDH+Age_at_ASCT+Sex+4;14	0.6715	0.6338

Abbreviation: ISS, the International Stage System; Diagnosis_to_ASCT, time from myeloma diagnosis to ASCT; LDH, Lactate Dehydrogenase; gep, gene expression profiling; Sky92, SkylineDx 92 high-risk gene model; 14;16, 14;20, 4;14 represent chromosome translocation.

Supplemental Table S9. Cox models' c-index of variables in ATM4S combine with 17p del in 1019 ASCT MM subgroup

Variables in Cox model	c-index of OS	c-index of PFS
ISS+Diagnosis_to_ASCT+Ferritin+Transferrin+LDH+Age_at_ASCT+Sex	0.6596	0.6257
ISS+Diagnosis_to_ASCT+Ferritin+Transferrin+LDH+Age_at_ASCT+Sex+17p del	0.6677	0.6331

Abbreviation: ISS, the International Stage System; Diagnosis_to_ASCT, time from myeloma diagnosis to ASCT; LDH, Lactate Dehydrogenase;

Supplemental Table S10. Cox models' c-index of variables in ATM4S combine with 13q del in 459 ASCT MM subgroup

Variables in Cox model	c-index of OS	c-index of PFS
ISS+Diagnosis_to_ASCT+Ferritin+Transferrin+LDH+Age_at_ASCT+Sex	0.6672	0.6298
ISS+Diagnosis_to_ASCT+Ferritin+Transferrin+LDH+Age_at_ASCT+Sex+13q del	0.6779	0.6379

Abbreviation: ISS, the International Stage System; Diagnosis_to_ASCT, time from myeloma diagnosis to ASCT; LDH, Lactate Dehydrogenase;

Supplemental Table S11. Cox models' c-index of variables in ATM4S combine with 1p del and 1q gain in 1154 ASCT MM subgroup

Variables in Cox model	c-index of OS	c-index of PFS
ISS+Diagnosis_to_ASCT+Fer	0.6638	0.6316

ritin+Transferrin+LDH+Age_at_ASCT+Sex		
ISS+Diagnosis_to_ASCT+Ferritin+Transferrin+LDH+Age_at_ASCT+Sex+1pdel	0.6693	0.6370
ISS+Diagnosis_to_ASCT+Ferritin+Transferrin+LDH+Age_at_ASCT+Sex+1q gain	0.6776	0.6432

Abbreviation: ISS, the International Stage System; Diagnosis_to_ASCT, time from myeloma diagnosis to ASCT; LDH, Lactate Dehydrogenase;

Supplemental figures

Supplement Figure S1. Survival curves of variables present in < 20% high risk myeloma.

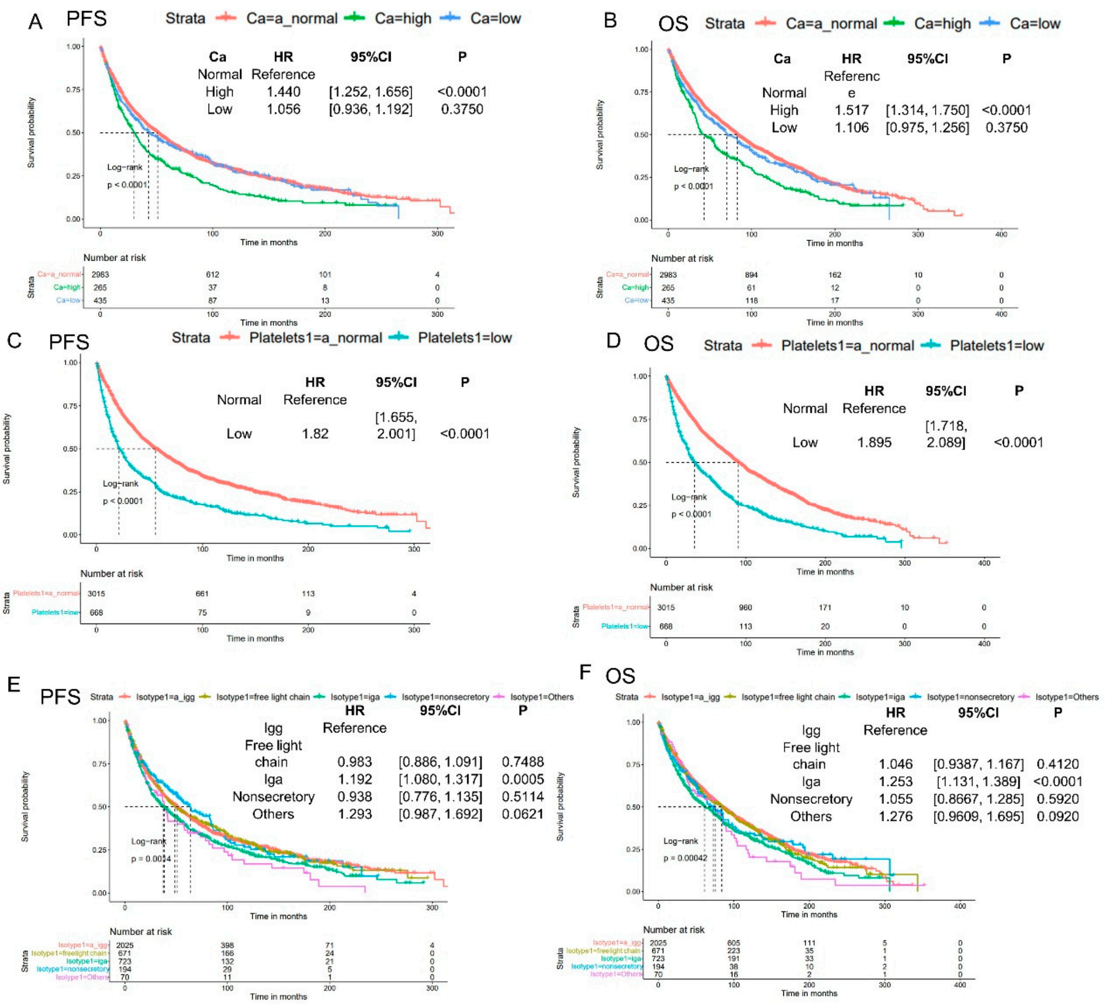

A, B. Survival curves in calcium high/normal/low groups. C, D. Survival curves in platelet high/low groups. E, F. Survival curves in different isotypes' subgroups. Survival curves were generated for different subgroups based on calcium levels (high/low/normal), platelet counts (normal/low), and Isotype groups. Notably, high calcium levels, low platelet counts, and IgA Isotype were associated with a relatively higher hazard ratio in terms of Progression-Free Survival (PFS) and Overall Survival (OS). These factors were only characteristic of a small proportion of the patient population.

Supplement Figure S2. 4-stage system by k-adaptive partitioning and adjustment.

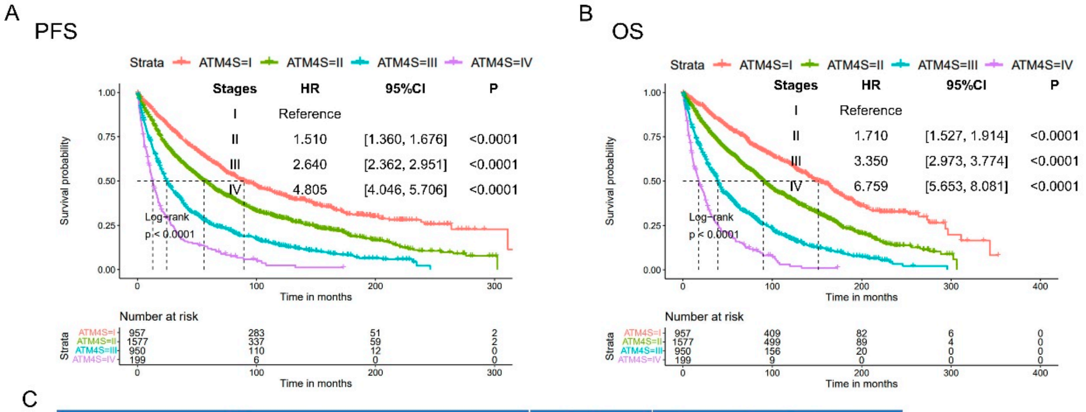

C

Stage systems	Border	Proportion of each stage
Original ATM4S defined by K-adaptive partitioning	I: [0, 1]<br>II: (1, 3]<br>III: (3, 5]<br>IV: (5, 7.5]	I: 26.0%<br>II: 42.8%<br>III: 25.8%<br>IV: 5.4%
Adjusted ATM4S	I: [0, 1]<br>II: (1, 2.5]<br>III: (2.5, 4]<br>IV: (4, 7.5]	I: 26.0%<br>II: 27.9%<br>III: 30.9%<br>IV: 15.2%

A, B. Progression free survival and overall survival of original ATM4S defined by K-adaptive partitioning. C. Border of original ATM4S and adjusted ATM4S model and patients' proportion in each stage.

Supplement Figure S3. Assessment of model generalization (Overall survival).

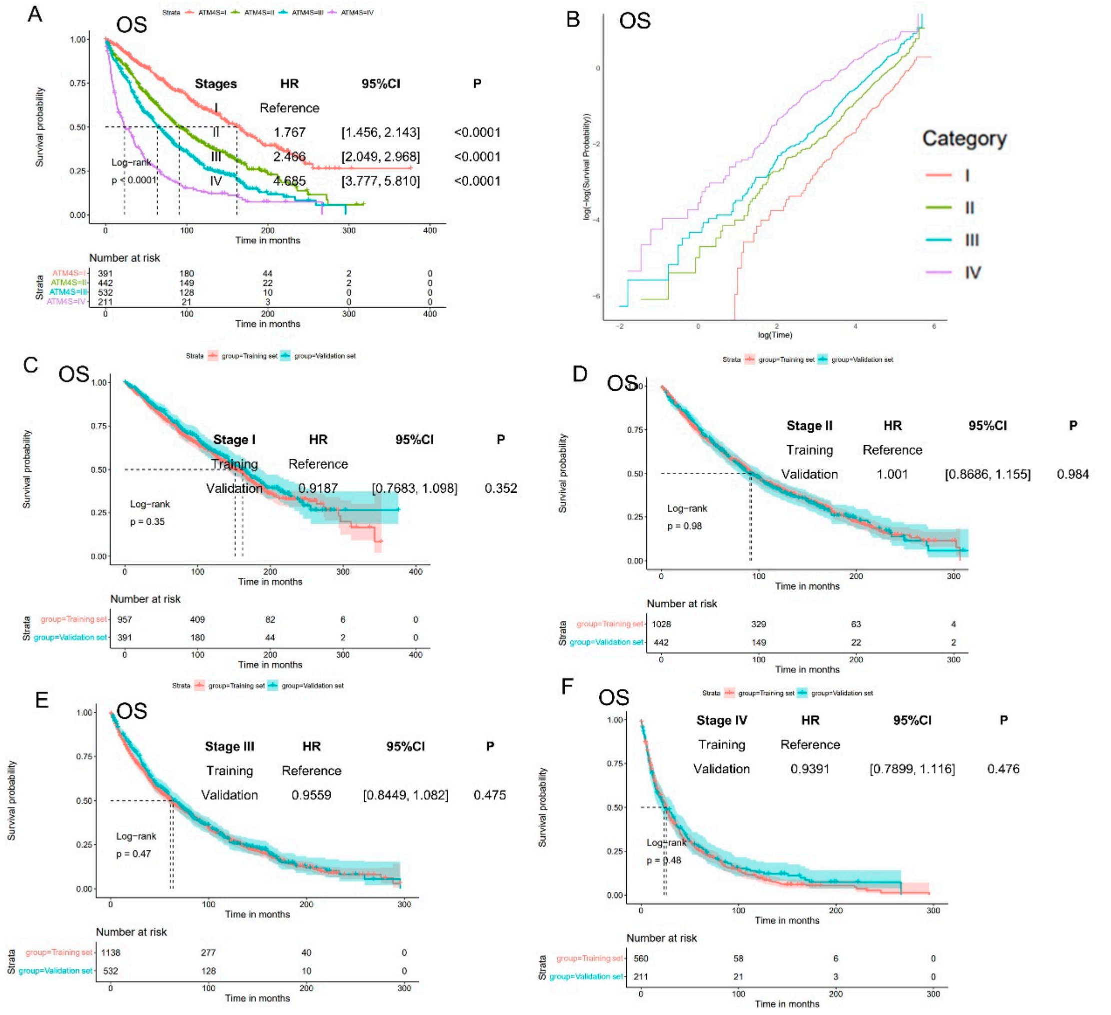

A. Progression free survival curves of ATM4S in UAMS validation cohort. The 4-stage system demonstrates an anticipated distinction in prognosis. B. Log-negative-log plot of different stages. The survival curves of each group exhibit a parallel pattern, suggesting that they meet the proportional hazard assumption. C-F. Comparison of overall survival curves for each stage in both the training and validation datasets. No statistically significant differences were observed within each stage, suggesting a consistent effect of the stage system in both datasets.

Supplement Figure S4. ATM4S in different clinical trial subsets.

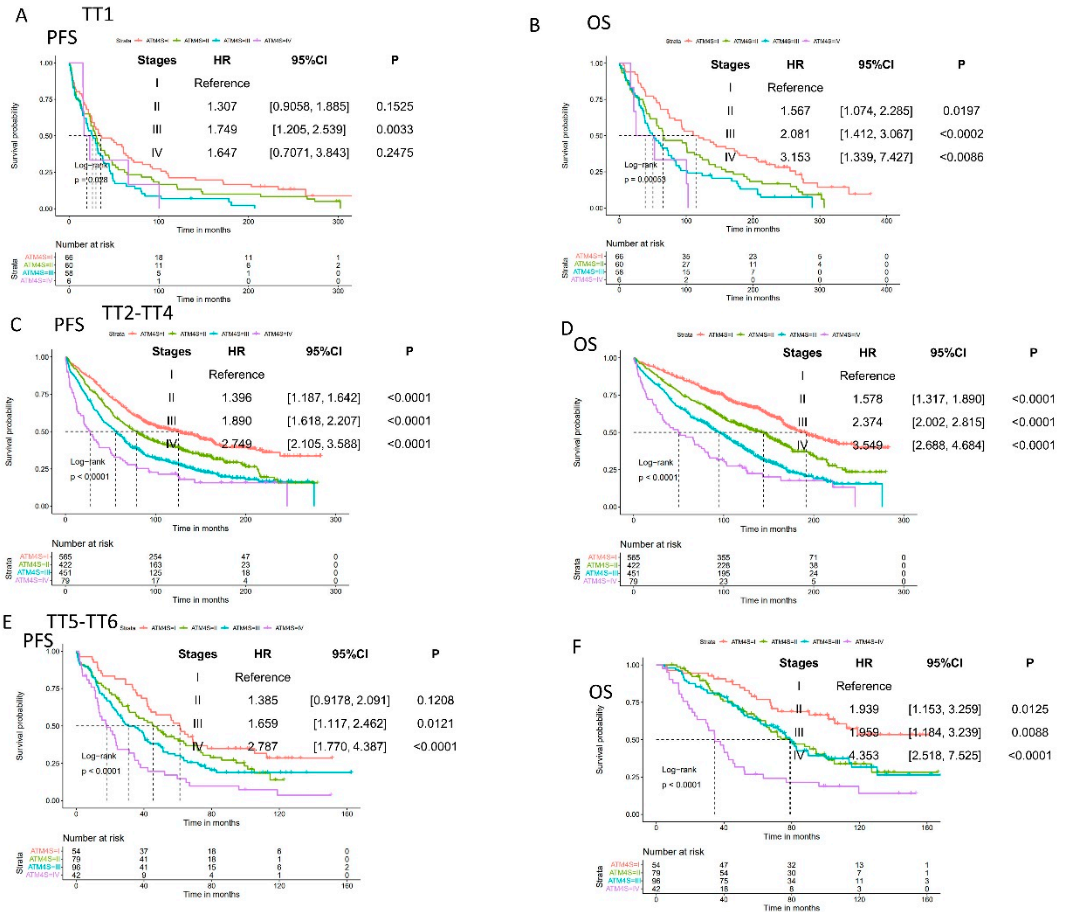

A, B. Progression free and overall survival curves of ATM4S subgroups in total therapy 1 subgroups. C, D. Progression free and overall survival curves of ATM4S subgroups in total therapy 2, 3, 4 subgroups. E, F. Progression free and overall survival curves of ATM4S subgroups in total therapy 5, 6 subgroups. Detailed information of total therapy can be seen in supplementary Table S7.

Reference

1. Zhan F, Hardin J, Kordsmeier B, Bumm K, Zheng M, Tian E, et al. Global gene expression profiling of multiple myeloma, monoclonal gammopathy of undetermined significance, and normal bone marrow plasma cells. Blood. 2002 Mar 1;99(5):1745-57.

2. Zhan F, Huang Y, Colla S, Stewart JP, Hanamura I, Gupta S, et al. The molecular classification of multiple myeloma. Blood. 2006 Sep 15;108(6):2020-8.

3. Hanamura I, Stewart JP, Huang Y, Zhan F, Santra M, Sawyer JR, et al. Frequent gain of chromosome band 1q21 in plasma-cell dyscrasias detected by fluorescence in situ hybridization: incidence increases from MGUS to relapsed myeloma and is related to prognosis and disease progression following tandem stem-cell transplantation. Blood. 2006 Sep 1;108(5):1724-32.

4. Shaughnessy J, Jacobson J, Sawyer J, McCoy J, Fassas A, Zhan F, et al. Continuous absence of metaphase-defined cytogenetic abnormalities, especially of chromosome 13 and hypodiploidy, ensures long-term survival in multiple myeloma treated with Total Therapy I: interpretation in the context of global gene expression. Blood. 2003 May 15;101(10):3849-56.

5. Xiong W, Wu X, Starnes S, Johnson SK, Haessler J, Wang S, et al. An analysis of the clinical and biologic significance of TP53 loss and the identification of potential novel transcriptional targets of TP53 in multiple myeloma. Blood. 2008 Nov 15;112(10):4235-46.

6. Chang H, Qi C, Yi QL, Reece D, Stewart AK. p53 gene deletion detected by fluorescence in situ hybridization is an adverse prognostic factor for patients with multiple myeloma following autologous stem cell transplantation. Blood. 2005 Jan 1;105(1):358-60.

7. Greipp PR, San Miguel J, Durie BG, Crowley JJ, Barlogie B, Blade J, et al. International staging system for multiple myeloma. Journal of clinical oncology : official journal of the American Society of Clinical Oncology. 2005 May 20;23(15):3412-20.

8. D'Agostino M, Cairns DA, Lahuerta JJ, Wester R, Bertsch U, Waage A, et al. Second Revision of the International Staging System (R2-ISS) for Overall Survival in Multiple Myeloma: A European Myeloma Network (EMN) Report Within the HARMONY Project. Journal of clinical oncology : official journal of the American Society of Clinical Oncology. 2022 Oct 10;40(29):3406-18.

9. Durie BG, Harousseau JL, Miguel JS, Blade J, Barlogie B, Anderson K, et al. International uniform response criteria for multiple myeloma. Leukemia. 2006 Sep;20(9):1467-73.

10. Shaughnessy JD, Jr., Zhan F, Burington BE, Huang Y, Colla S, Hanamura I, et al. A validated gene expression model of high-risk multiple myeloma is defined by deregulated expression of genes mapping to chromosome 1. Blood. 2007 Mar 15;109(6):2276-84.

11. Shaughnessy JD, Jr., Qu P, Usmani S, Heuck CJ, Zhang Q, Zhou Y, et al. Pharmacogenomics of bortezomib test-dosing identifies hyperexpression of proteasome genes, especially PSMD4, as novel high-risk feature in myeloma treated with Total Therapy 3. Blood. 2011 Sep 29;118(13):3512-24.

12. Kuiper R, Broyl A, de Knegt Y, van Vliet MH, van Beers EH, van der Holt B, et al. A gene expression signature for high-risk multiple myeloma. Leukemia. 2012 Nov;26(11):2406-13.

13. Zhang Z, Gayle AA, Wang J, Zhang H, Cardinal-Fernandez P. Comparing baseline characteristics between groups: an introduction to the CBCgrps package. Ann Transl Med.

2017 Dec;5(24):484.
